# Experimental and Numerical Analysis of the Cooling Performance of Water Spraying Systems during a Fire

**DOI:** 10.1371/journal.pone.0118306

**Published:** 2015-02-27

**Authors:** YaoHan Chen, ChungHwei Su, JoMing Tseng, WunJie Li

**Affiliations:** 1 Department of Fire Science, WuFeng University, Minxiong Township, Chiayi County, Taiwan; 2 Department of Safety, Health and Environmental Engineering, National Kaohsiung First University of Science and Technology, Kaohsiung City, Taiwan; 3 Institute of Safety and Disaster Prevention Technology, Central Taiwan University of Science and Technology, Taichung City, Taiwan; 4 Graduate School of Opto-Mechatronics and Materials, WuFeng University, Minxiong Township, Chiayi County, Taiwan; North China Electric Power University, CHINA

## Abstract

The water spray systems are effective protection systems in the confined or unconfined spaces to avoid the damage to building structures since the high temperature when fires occur. NFPA 15 and 502 have suggested respectively that the factories or vehicle tunnels install water spray systems to protect the machinery and structures. This study discussed the cooling effect of water spray systems in experimental and numerical analyses. The actual combustion of woods were compared with the numerical simulations. The results showed that although the flame continued, the cooling effects by water spraying process within 120 seconds were obvious. The results also indicated that the simulation results of the fifth version Fire Dynamics Simulator (FDS) overestimated the space temperature before water spraying in the case of the same water spray system.

## Introduction

### Numerical simulation on the performance of building service devices

Numerical simulation software can obtain abundant valuable findings with lower research and design costs and time as well as more flexible setting [1, 2, 3]. Some software packages include: JASMINE, STAR-CD, Phoenix, KAMELEON, CFX, FLUENT, SOFIE and FDS. For the building fire and smoke control with large investment amount and high level of difficuly, the numerical simulation analysis provides researchers and designers with excellent auxiliary functions [4, 5].

The international fire safety domain often uses Fire Dynamics Simulator (FDS) software as an analytical tool. The FDS uses LES as its main computation schema, describing the gas flow phenomenon driven by fire dynamism. The FDS software was developed by the Building and Fire Research

Laboratory of the National Institute of Standards and Technology (NIST); it analyzes the computational fluid dynamics (CFD) of fire heat flow. Since the first version of FDS was issued in February 2000, the FDS 5.0.0 was published in October 2007. The FDS 5.5.3 issued on October 29, 2010 was the fifth and final version. The formal FDS 6.1.0 of the sixth version issued on May 29, 2014 contained some updated items, e.g. Hydrodynamics and Turbulence, Species and Combustion, HVAC, and Radiation; it can simulate more items more accurately [6].

The FDS was commonly applied to the simulation and verification of smoke exhaust systems and evacuation systems for many buildings, as well as to the research on tunnel safety and industrial fires[7, 8]. Some studies were conducted to discuss the heat release rate of the material combustion and the validity of peformance-based design in buildings [9–12]. In recent years, some literatures analyzed the interaction of mist, smoke and ventilation in the fifth edition or older of the FDS software. [13–16].

### Fire suppression and cooling effect for water spray systems

The structure of a building will be severely damaged due to the expansion of the fire and the high temperature. According to the description of NFPA 15: Standard for Water Spray Fixed Systems for Fire Protection, the water spray system can extinguish general combustibles, such as paper, wood and textiles. It can prevent fire triggered by inflammable gases and inflammable liquid. In addition, differing from water-based systems, such as sprinkler systems and foam-water sprinkler systems, the water spray system has perfect protective effect on electrical fires, e.g. transformer, motor and cable tray fire [17]. According to the description of NFPA 502, when a tunnel is on fire, the water spray system can effectively protect the structure [18]. Especially in a vehicle tunnel with large fire load, the closed space results in the accumulation of hot gas. Some noted organizations have discussed the effect of sprinkler cooling equipments inside tunnels [19, 20].

### The accuracy of the numerical simulation

From October 2007 to May 2014, many large buildings and factories, as well as some special buildings using the performance-based design method in Taiwan employed FDS as the design tool. A few cases implemented full-scale validation; most of the official fire protection organs used the FDS simulation results as the unique judgment criteria of building fire safety [21, 22]. This study discusses the cooling effect of water spray system on the designated space. The sprinkling cooling effect of the water spray system on burning wood is tested, and the results are compared with those of a numerical simulation. The cooling effectiveness of water spray system is discussed, and the difference between FDS Ver.6 released on May 29, 2014 and previous versions is found in the comparison between experimental and numerical simulation results.

### Heat transfer between spraying droplets and air

The Reynolds number for a spraying droplet with various diameters i is expressed as:

$$\text{Re}(i)\;=\frac{u•i}{\nu }$$ (1)

where u and υ are respectively the velocity and the kinematic viscosity of the air. The Nusselt number is defined by the ratio of convection heat transfer to conduction heat. The inner diameter of the spray droplets i is [23]:

$$Nu(i)\;=2\text{+[0.4 }{\text{Re(i)}}^{\text{0.5}}\text{+0.06 }{\text{Re(i)}}^{\text{2/3}}\text{] }{\text{Pr}}^{\text{0.4}}$$ (2)

Pr is the Prandtl number of the air, and taken as 0.7. The energy conservation equation is expressed as:

$$m(i)\;C_{p}\frac{\text{d}T{\left(i\right)}_{w}}{\text{d}t}=h\left(i\right)S\left(i\right)\left[T-T{\left(i\right)}_{w}\right]$$ (3)

where m(i) and T(i)w are the mass fraction and the temperature of droplets with diameter I, respectively. S(i) is the external surface area of droplets, and T is the ambient temperature.

## Experimental Method

### Experimental process

The dimension of the test room is 8(m)×8(m)×6(m). The FDS program set the temperature measuring point in the same position as the experiment. Table 1 shows the description of codes of the experimental case and FDS simulation case, as well as the set situation.

**Table 1:** Description of experiment and numerical simulation cases.

FDS simulation	Case symbol	Scenario description
Version 5	FDS 5	Woodpile burning / without water spraying
	FDS 5 spk	water spraying@120 seconds
	FDS 5 spk-n	water spraying optioned but inactive
Version 6	FDS 6	without water spraying
	FDS 6 spk	water spraying@120 seconds
	FDS 6 spk-n	water spraying optioned but inactive
Experimental test	W-A2	without water spraying
	W-A2 spk	water spraying@120 seconds

This study compares the flame profile and temperature change in the wood combustion process in the results of experiment and numerical simulation (Ver.5 and Ver.6). The space cooling effect was tested by a water spray system. The measuring instruments included a thermocouple measurement device and infrared thermal imager to record the temperature change.

### Layout of experimental equipment

**Fig 1:**
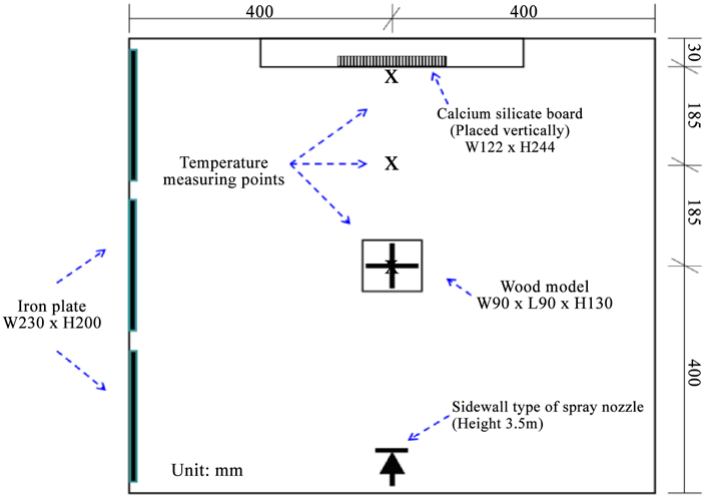
Layout of experimental equipments.

**(1) Woodpile model**. Fig. 1 shows the layout of the experimental site. The wood model for this combustion refers to the extinguishing capabilities of the “*Approval Directions for Fire Extinguisher*” published in July 2013 in Taiwan, in testing the extinguishing ability of fire extinguishers [24].

**Fig 2:**
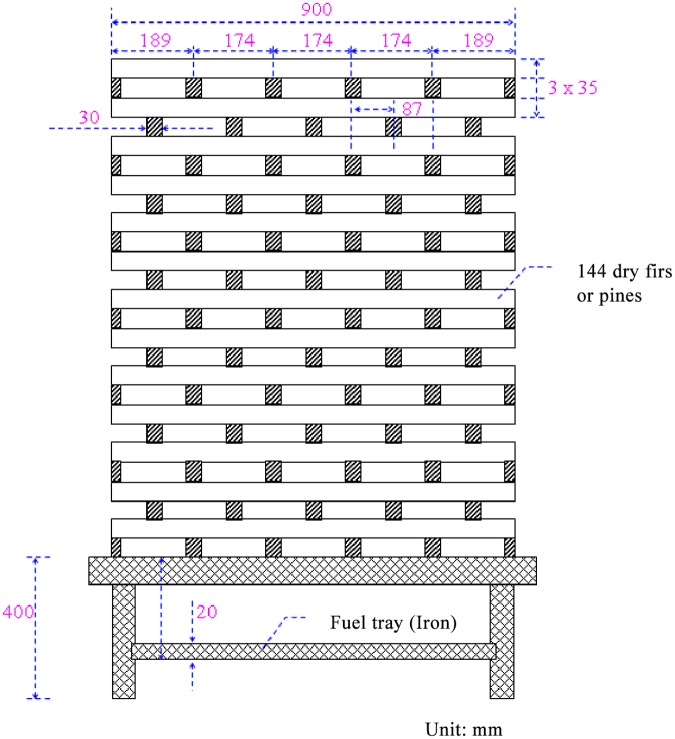
Wood model (for experiment).

The experiment adopted the A-2 model, and the overall structure was composed of a 30mm×35mm×900mm stack. There were 144 wood pieces, the total surface area was about 16m^2^, and the weight was about 54kg. The configuration height of the woodpile was from 0.4 to 1.3 m, as shown in Fig. 2.

**(2) Water spray system**. The water spray system used in this study was equipped with a sidewall spray nozzle. In consideration of the extinguishing and cooling effectiveness on burning wood, the nozzle was mounted 3.5m above the floor. The sprinkler was started up 120 seconds after ignition, as shown in Table 2.

**Table 2:** The spray nozzle type.

Sprinkler type	Sidewall
Water flow rate	380 L/min
Height of the device	3.5 m
Activated time to spray	120 seconds after ignition

**Fig 3:**
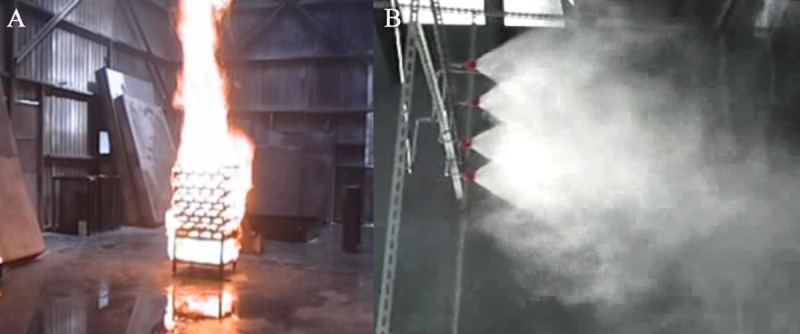
A: Combustion conditions of wood. B: Water spraying situation on the site.

**Fig 4:**
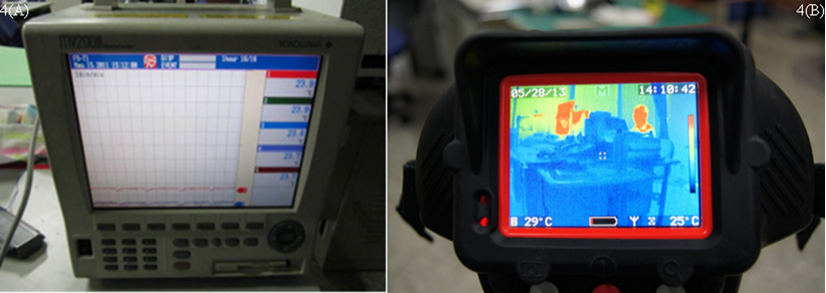
A: Thermocouple measurement device. B: Infrared thermal imager.

**(3) Temperature measurement device and infrared thermal imager**. Fig. 3(A) shows the combustion of wood in the testing process. The sprinkling of the water spray system is shown in Fig. 3(B). In order to analyze the space temperature, the K-type thermocouple was mounted to record the temperature of combustion. The YOGOKAWA (MV200 type) recorder stored the temperature value, as shown in Fig. 4(A). The recorder recorded the temperature change values. The position of the thermocouple measuring point is straight above the fire source, 4.6m from the floor, referring to the research method of Hietaniemi [25]. The infrared thermal imager recorded the temperature change in the overall space; the temperature image was used to analyze the experimental result, as shown in Fig. 4(B).

## Numerical Analysis Method

### The description of the Fire Dynamics Simulator

Various governing equations are used to calculate the velocity, pressure, temperature and concentration values accurately represented by grid points in various positions of the fire. General fire simulation uses the “Zone Model” and “Field Model” for simulation. For buildings in complicated shapes, the Field Model can accurately simulate the situation. At present, the Field Model is mostly used for simulations [26, 27].

### Fire scenario

**(1) Grid Analysis**. The FDS grid design must pay attention to both the efficiency and accuracy of the simulation. These two items are the main considerations in the simulation process [28, 29]. In terms of the evaluation of the optimum grid, this case uses a fire source characteristic diameter computing mode to analyze the optimum grid size at the maximum heat release rate. McCaffery proposed using the minimum length scale of fire plume as the characteristic fire diameter D * to determine the grid size [30]:

D*=[Q˙ρ∞ C∞ T∞ g]25 (4)

where $\dot{Q}$: total heat release rate, kW

*C*_∞_: air specific heat, kJ/kg-K

g: gravitational acceleration, m/sec^2^

*ρ*_∞_: air density, kg/m^3^

*T*_∞_: ambient temperature, K

**Fig 5:**
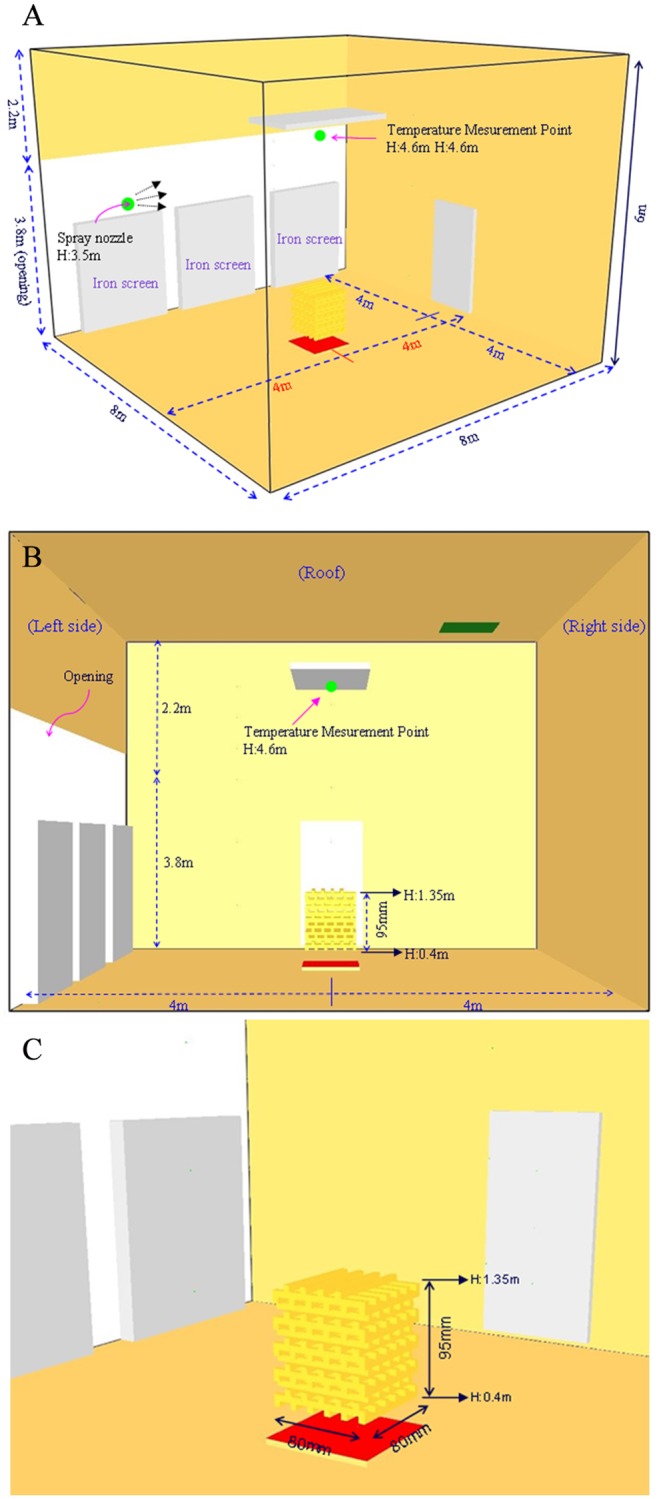
A: Three-dimensional isometric view of the simulation model. B: Sectional view of the simulation model. C: Isometric view of wood model.

When the model set grid size is about one tenth that of the characteristic diameter (D*), the LES simulated time average axis velocity and temperature match the experimental regression equation of McCaffery [30]. Figs. 5(A) and (B) show the overall dimension and layout of the model space. The top and right of Fig. 5(A) show the solid wall. There is an open plane to the left of the figure. The iron curtain prevents the hot gas from flowing out of the open surface.

The heat release rate (HRR) of the fire source was about 2.2MW. The model analysis uses nonuniform grid layout. The overall grid size is 0.1m, meeting the calculated value smaller than 0.1D *. The grid encryption is 0.05m in the range of 2m×2m×6m near the fire source center.

**(2) Wood model in numerical simulation**. Considering the computing performance and simulation accuracy of computer equipment, this study uses FDS Ver.5 and 6 to simulate the wood combustion process. The grid size near the fire source uses an encrypted grid, which is 5.0cm. The combustion considers the wood heat release rate (HRR), as shown in Table 3.

**Table 3:** Properties of wood.

	Numerical Simulation	Experiment
Size	50×50×800mm	30×35×900mm
Heat Release Rate Per Unit Area (HRRPUA)	175 kW/m^2^	
Density	369.6 kg/m^3^	
Combustion heat	17,900 kJ/kg	
Ignition temperature	384°C	
Volume	0.002 m^3^/each	0.000945 m^3^/each
Surface Area	0.165 m^2^/each	0.1191 m^2^/each
The total number	104 sticks	144 sticks
The total surface Area	17.16 m^2^	17.1504 m^2^

**Fig 6:**
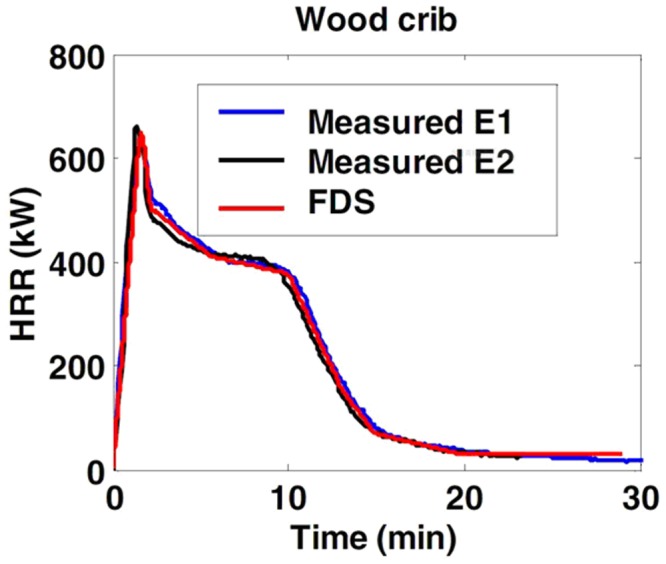
Heat release rate (HRR) curve of the wood model [31]. (Rinne et al., 2007).

The size of each wood piece is limited to the grid size during simulation, so it is defined as 50x50x800mm. As the heat release rate of the numerical simulation should be consistent with that of the experiment, the total surface area of experimental wood is 17.15m^2^. The surface area of one wood piece for simulation is 0.165 m^2^, so the total number of wood pieces is about 104. The wood pieces are stacked in layers crosswise; the 104 pieces are arranged orderly. The spread geometry is shown in Fig. 5(C). The assumptions of numerical simulation are shown in Table 3. For the wood HRR combustion curve, relevant setup parameters refer to the test report of VTT (Valtion Teknillinen Tutkimuskeskus) in 2007 [31], as shown in the curve in Fig. 6.

## Results and Discussion

### Heat release rate of the wood model in the numerical simulation

**Fig 7:**
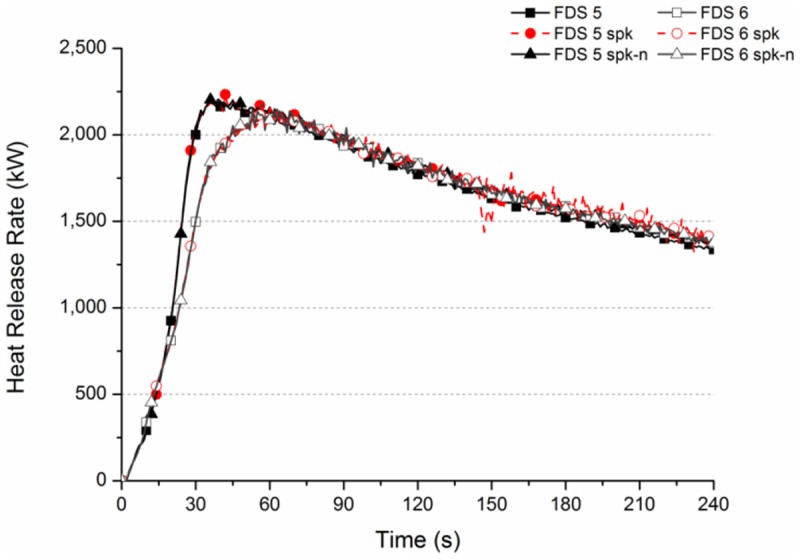
Heat release rate curve of numerical simulation cases.

The results of the numerical simulation are analyzed as follows. The HRR curves of six simulation models are shown in Fig. 7, and means that whether or not there is sprinkling, the HRR curves of the two versions overlap each other considerably. The combustion mode of wood is set as the phenomenon of natural spread, not a compulsive burning rate. The figure shows that the maximum HRR value of wood combustion is about 2.0 to 2.2 MW. The maximum HRR value of FDS Ver.5 is slightly different from that of Ver.6, with a little difference in the time.

The simulation result shows that the HRR increases when the combustion begins. The three cases of FDS Ver.5 show that regardless whether or not there is sprinkling, the maximum heat release value occurs at 30 s to 40 s, and then gradually decreases. The Case FDS 5 spk decreases slightly at 150 s. The HRR is about 1.75 MW at 120 s. The three cases of FDS Ver.6 show that the maximum value occurs between 50 s and 60 s. It decreases gradually as time goes on, about 1.75 MW at 120 s.

Fig. 7 shows that whether or not there is sprinkling, the HRR curves of the two versions overlap each other considerably, meaning the HRR changes slightly due to the sustained combustion of the fire source in the simulation. The cooling effect of water spray system can be known by analyzing the space temperature change.

### Distortion calculation while starting the spraying function in FDS Ver.5

Table 4 shows the temperature values at 4.6m straight above the fire source in all cases at different time points; later changes in various cases will be described in Table 4.

**Table 4:** Temperature at different times for various cases.

Time (s)	Experiment		FDS Version 5*			FDS Version 6*		
	W-A2	W-A2 spk	FDS 5	FDS 5 spk-n	FDS 5 spk	FDS 6	FDS 6 spk-n	FDS 6 spk
30	274	215	978	970	984	393	393	366
60	500	485	1018	651	645	388	388	372
90	443	495	1006	702	691	311	311	317
115	500	511	975	688	543	379	379	380
125	420	443	999	642	530	321	321	353
150	294	135	765	561	113	283	283	173
180	257	88	697	522	45	284	284	178
210	216	74	705	523	29	242	242	178
240	232	72	581	471	26	270	270	163

* The average within 10 s.

According to the simulation results, the simulated phenomenon of FDS Ver.5 has a strange characteristic. If the simulated case has water spray equipment which does not spray water, the simulation result may be distorted.

In practice, if the water spray equipment does not spray water, the space temperature change in Case FDS 5 spk-n should be identical with the Case FDS 5 without water spray system. Since the water is not sprayed out after all, the space is not cooled by water. Theoretically, the two temperature curves of Case FDS 5 and Case FDS 5 spk-n are supposed to be identical.

**Fig 8:**
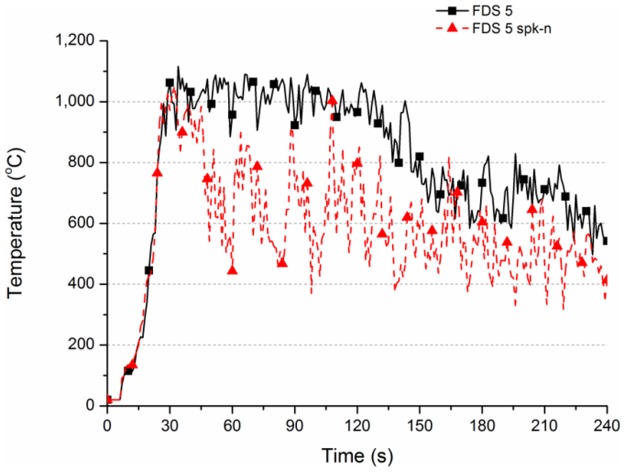
Temperature variation for different settings (for FDS Ver.5).

However, according to Table 4 and Fig. 8, the two curves are unexpectedly different. According to the result of Case FDS 5 spk-n, the calculated temperature decreases obviously if the simulated case has a water spray system. At 60 s, the maximum value of Case FDS 5 is 1018°C, that of Case FDS 5 spk-n is 651°C. At 90 s, the maximum value of Case FDS 5 is 1006°C, and that of Case FDS 5 spk-n is 702°C.

**Fig 9:**
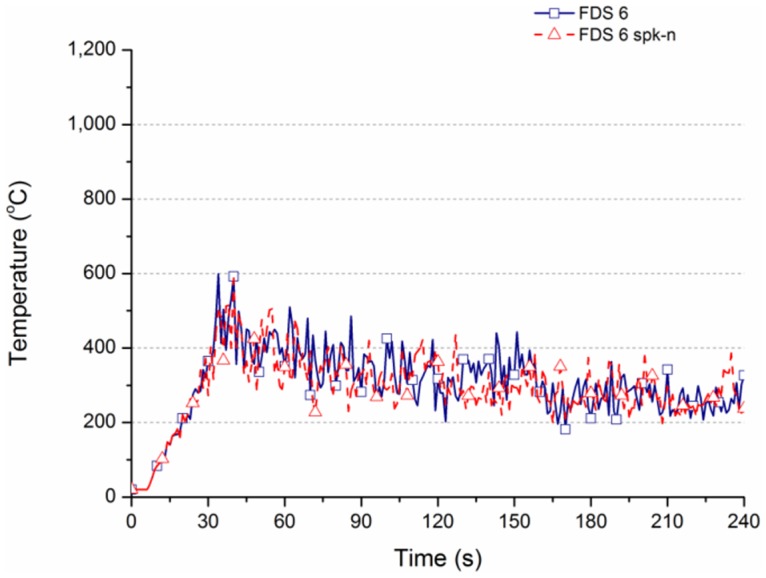
Temperature variation for different settings of FDS Ver.6.

Comparatively, the simulation result of FDS Ver.6 is undistorted. According to Table 4, the values of Case FDS 6 and Case 6 spk-n are almost identical. Fig. 9 shows that the overlap of two temperature curves is very obvious. Apparently, the space temperature calculator system has been improved in the FDS Ver.6. Compared with Fig. 8, the curve in Fig. 9 shows the maximum temperature value is 600°C at 35 s to 45 s. The two cases show that the maximum value of Case FDS 6 is 388°C at 60 s, while that of Case FDS 6 spk-n is 372°C. At 90 s, the maximum value of Case FDS 6 is 311°C, while that of Case FDS 6 spk-n is 317°C. The calculated value is 100 to 180°C = lower than the actual value.

### The cooling effect of the fire space in the numerical simulation

**Fig 10:**
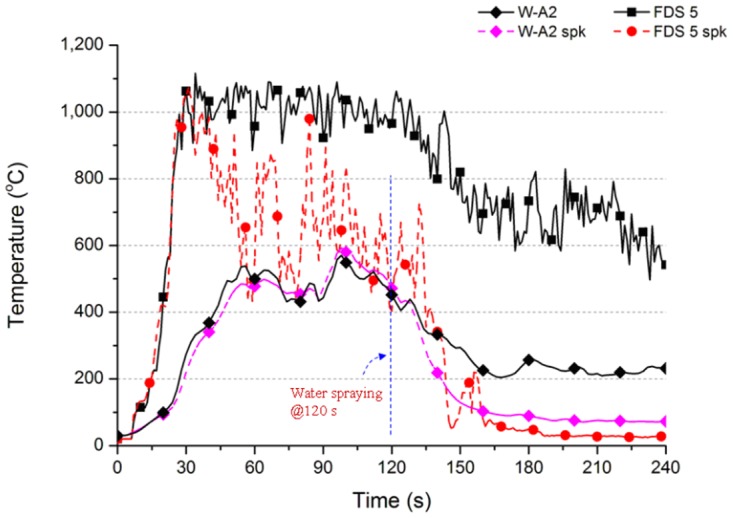
Temperature variation of experimental and numerical simulation cases (for FDS Ver.5).

The cooling effect of FDS Ver.5 and Ver.6 was simulated in this study. As the research subject was the cooling effect, the experimental results of Case W-A2 and Case W-A2 spk were used as the comparison base. The simulation results of space temperature values when the water spray system was actuated were recorded as the comparative value of the cooling effect of the water spray system on actual wood combustion. The temperature varied at 4.6m above the fire source in the simulation cases; the two experimental cases are shown in Figs. 10 and 12.

For the actual combustion of wood in Case W-A2, Fig. 10 shows that the fire behavior increased gradually before 60 s, and the temperature remained at 450 to 550°C before sprinkling. The curve of Case W-A2 spk shows that the temperature began to rise at 60 s, and the temperature remained stable. The maximum temperature of about 600°C occurred in the two cases between 90 s and 105 s. When the sprinkling began at 120 s, the space temperature dropped obviously. The temperature decreased to about 88°C at 180 s. The temperature decreased to about 72°C at 240 s. If the water is not sprayed, the temperature decreased to about 232°C at 240 s.

The numerical simulation result was analyzed; in 120 s before sprinkling, the simulation result of FDS Ver.5 shows that the temperature was apparently high. The maximum value occurred ahead of time. The temperature of Case FDS 5 was higher than 1000°C after 30 s. The temperature was 1018°C at 60 s and 1006°C at 90 s. The temperature began to fall at about 130 s. The temperature was still 581°C at 240 s. The temperature of Case W-A2 was about 232°C at 240 s. The difference between the experimental and simulation results is 349°C.

Before sprinkling at 120 s, the temperature change of Case FDS 5 spk was quite different from Case FDS 5, but similar to the trend of Case FDS 5 spk-n in Fig. 8. The result validates the aforesaid statement; the simulation result of FDS Ver.5 may be distorted on certain conditions. At 30 s, Case FDS 5 spk had a high temperature of 987°C. The temperature fell drastically between 30 s and 120 s. The temperature range was about 500 to 1000°C. When the sprinkling began at 120 s, the temperature began to decrease at 130 s. Table 4 and Fig. 10 show that the temperature decreased from 543°C to 26°C. The cooling effect was good, but lower than the 72°C of Case W-A2 spk of actual wood combustion.

The results show two problems in simulating the cooling effect of water spray system in FDS Ver.5. First, without a water spray system, the calculated value of space temperature is relatively high: the actual value is 600°C, but the calculated value is 1100°C. The other problem is that the cooling effect of the system is overrated, so that the final space temperature is relatively low. The latter misdirection may result in excessively optimistic designers, so that less water is sprayed. Unfortunately, less sprinkling amount cannot reduce the actual space temperature to a safer low temperature, so the space remains at a high temperature.

**Fig 11:**
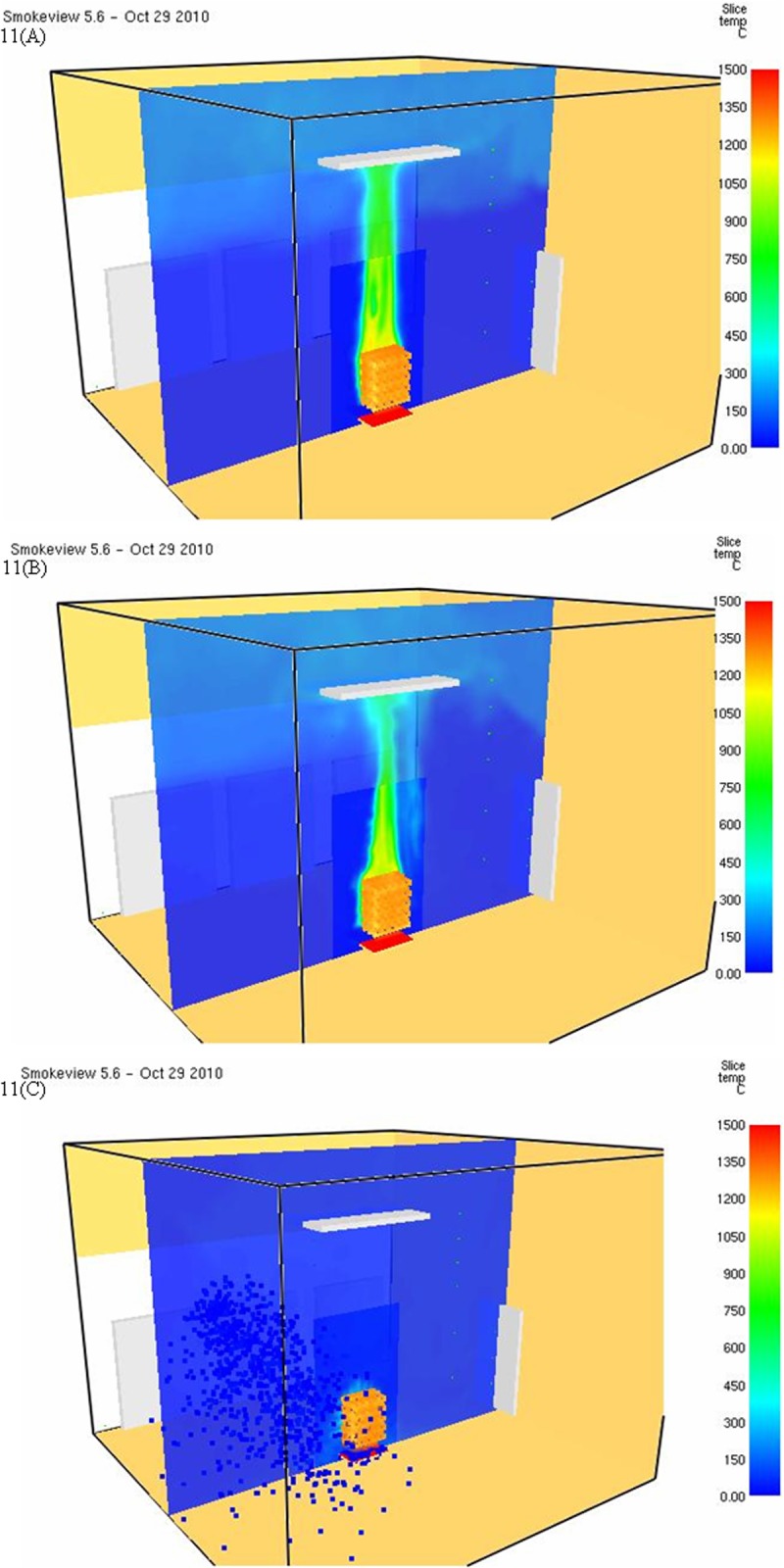
A: Temperature distribution of simulation results(Case FDS 5@180 s). B: Temperature distribution of simulation results(Case FDS 5 spk-n @180 s). C: Temperature distribution of Simulation results(Case FDS 5 spk @180 s).

Figs. 11(A) to (C) show the space temperature profile of different models by FDS Ver.5. This study extracted the combustion condition at 180 s to compare the temperature distribution. Figs. 11(A) and (B) show the temperature distribution of Case FDS 5 and Case FDS 5 spk n. As the space was not sprayed in the two cases, theoretically, the temperature distribution should be identical. Fig. 11(B) shows that the temperature distribution of Case FDS 5 spk n is low. For example, the high-temperature zone in the ceiling is obviously smaller, and the high temperature fire plume is smaller; these phenomena prove that the cooling effect of FDS Ver.5 on simulated space may be distorted. Fig. 11(C) shows the temperature distribution of Case FDS 5 spk. It is observed that the temperature decreases after sprinkling; for example, the high temperature fire plume almost disappears.

**Fig 12:**
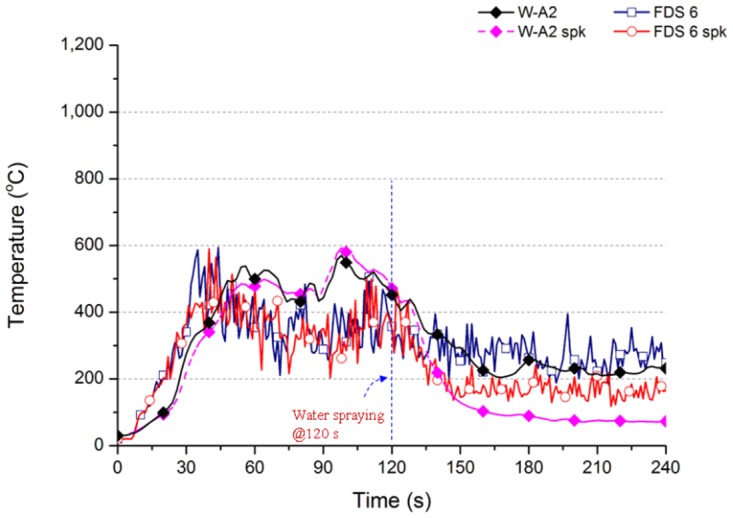
Temperature variation of experimental and numerical simulation cases (for FDS Ver.6).

Comparatively, the simulation results of FDS Ver.6 are consistent, and close to the actual result. Fig. 12 compares the numerical simulation and actual combustion results. Case W-A2 and Case W-A2 spk of actual wood combustion have the maximum temperature of 600°C at 95 to 105 s. At 120 s before sprinkling, the simulation result of FDS Ver.6 shows that the temperature change is similar to the actual value. The temperature curves of Case FDS 6 and Case FDS 6 spk overlap each other well. The maximum temperature value of Case FDS 6 before sprinkling is about 600°C at 35 s. The temperature stays at about 300 to 400°C between 30 s and 120 s. The temperature begins to fall after 110 s. The temperature is 284°C at 180 s, and 270°C at 240 s. The temperature of Case W-A2 is about 232°C at 240 s. The experimental and simulation results are close to each other: the difference is 38°C.

The temperature change of Case FDS 6 is slightly different from Case FDS 6 spk before 120 s. The temperature is 388 and 372°C, respectively, at 60 s. The temperature is 311 and 317°C, respectively, at 90 s. Fig. 9 shows the temperature of Case FDS 6 spk-n is 388 and 311°C, respectively, at 60 s and 90 s, meaning the simulation results of space temperature are consistent, which differs from FDS Ver.5. The temperature falls immediately after sprinkling at 120 s. Fig. 12 shows the temperature decreases from 500°C to 163°C, which is higher than the 72°C of Case W-A2 spk of actual wood combustion.

**Fig 13:**
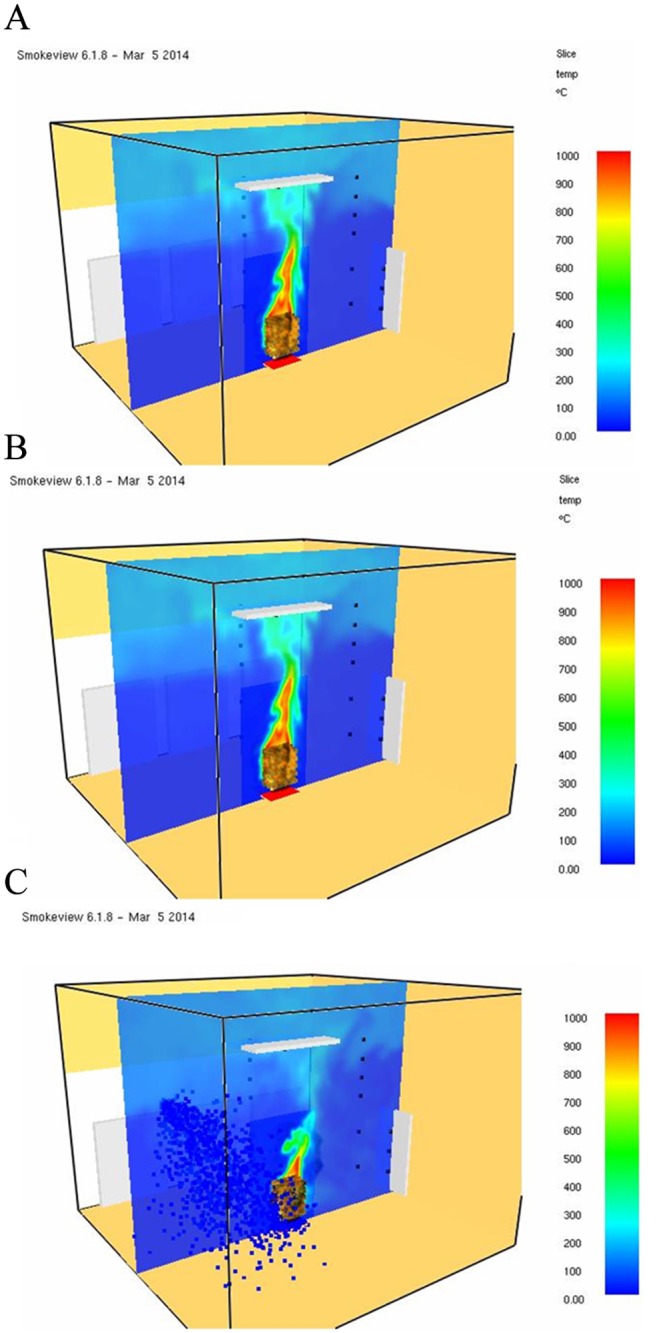
A: Temperature distribution of simulation results(Case FDS 6@180 s). B: Temperature distribution of simulation results(Case FDS 6 spk-n @180 s). C: Temperature distribution of simulation results(Case FDS 6 spk @180 s).

Figs. 13(A) to (C) show the space conditions of different models by FDS Ver.6; the distribution at 180 s is used for comparison. Figs. 13(A) and (B) show the temperature distribution of Case FDS 6 and Case FDS 6 spk n. The space is not sprinkled in the two cases; it is observed that the temperature distributions are almost identical, for example, the high-temperature zone size in the ceiling, or the distribution direction of heat current. The shapes of high temperature fire plumes are also similar to each other. The aforesaid figures show that FDS Ver.6 has consistent results of space cooling simulation. The temperature distribution of Case FDS 6 spk with sprinkling is shown in Fig. 13(C). It is observed that the high temperature in the ceiling is reduced a lot; the space is clearly cooled.

### The cooling effect of the fire space in an experimental test

**Fig 14:**
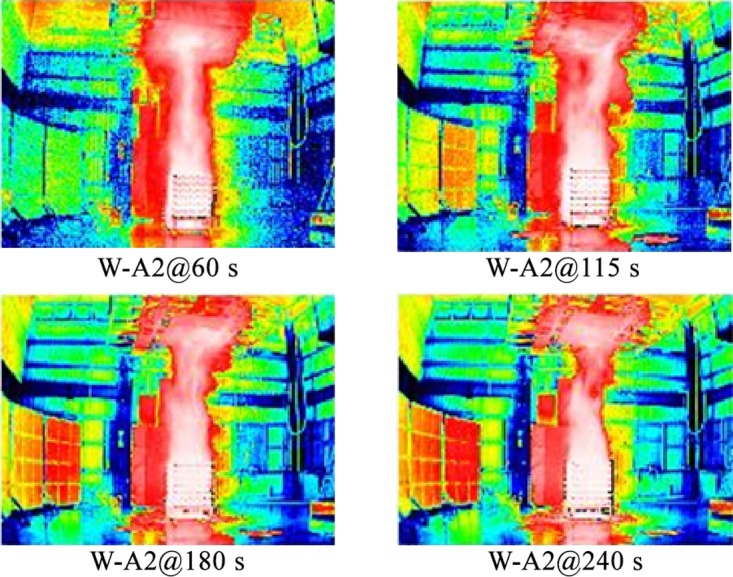
Temperature distribution of experiment results (for Case W-A2).

**Fig 15:**
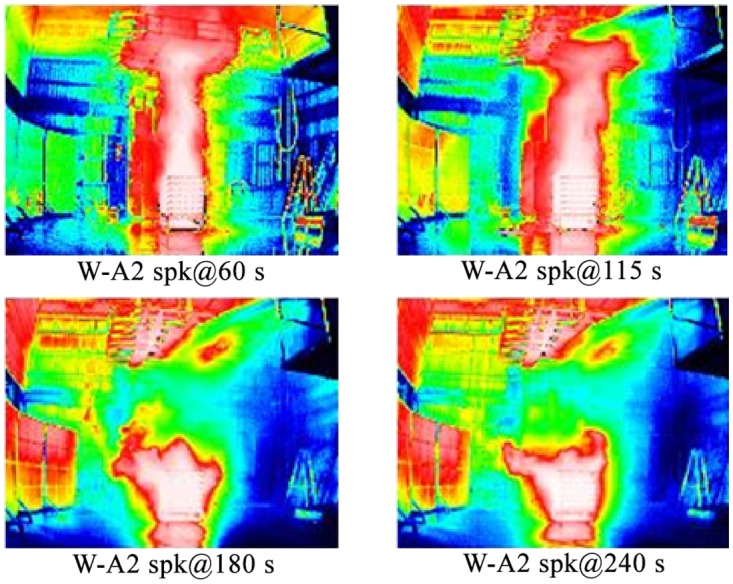
Temperature distribution of experiment results (for Case W-A2 spk).

In terms of the experiment, Figs. 14 and 15 show the record of infrared thermal imager. The red part represents high temperature, and the blue part represents low temperature. The experiment includes Case W-A2 of sustained wood combustion and Case W-A2 spk of sprinkling after 120 s of combustion. The space temperature profile in different periods is recorded.

When the wood is ignited, and the water spray system of Case W-A2 and Case W-A2 spk has not started up before 120s, there is no significant difference in the temperature distribution of the two cases at 60 s and 115 s. Figs. 14 and 15 show that the flame contacts the ceiling directly, so that the ceiling is red. In Fig. 15, when the sprinkling spray was activated at 120 s, the flame shape was obviously destroyed. The flame of Case W-A2 spk apparently diminishes after 180 s. It represents the effective cooling on the flame after the sprinkling. The space is apparently cooled. Finally, the flame is suppressed at 240 s.

The two figures clearly show the variation of overall space temperature with time. It is observed that the flame continues after the water is sprayed, but the cooling effect is obvious.

The results showed the divergence between numerical and experimental measurements. The temperatures in simulation cases were obviously higher than these ones in experiments. These trends in this study are similar to the results of Blanchard et al. studied on water mist systems[16]. The heat transfer mechanisms in the real fires are not only involved in the interaction between droplets and hot gases but also the vaporization of droplets. Moreover, the heat absorbed by droplets and radiative attenuation by vapor should not be ignored. The relevant heat transfer mechanisms in FDS are suggested to review to accurately simulate the cooling effect of water spray systems.

## Conclusions

The water spray system is one of the effective extinguishing and protection systems in confined or unconfined spaces when a fire occurs. NFPA 15 and 502 have suggested respectively that the factories or vehicle tunnels install water spray systems to protect their equipments and structures. Many large constructions have used the fifth version FDS to conduct the performance-based fire safety design in Taiwan.

The water spray system was tested in this study. The results showed that although the flame continued after the water spray, the cooling effect was obvious. For the numerical analysis, when the function of water spray system was optional but inactive, the simulated temperature value of the fifth version FDS significantly differed from the actual value, so that the simulation may be distorted.

The results showed that the simulation results of the fifth version FDS overestimated the space temperature before sprinkling in the case of the same water spray system. The actual temperature was 500°C, but the calculated temperature value was 975°C. There was a slight underestimation in simulation results of the sixth version FDS, but the difference was acceptable. The simulation result after sprinkling of the fifth version FDS showed that the temperature declined too fast; in comparison, the actual value was 72°C and the calculated value was 26°C. The calculated temperature in the sixth version FDS was higher, and the value was 163°C. Moreover, the results displayed that the simulated temperature value in the fifth version was significantly different from the actual value when the water spraying was optional but inactive. The simulation result of the fifth version FDS may reflect optimistic designers, as less water was sprayed, which is disadvantageous to space cooling. The results of this study can serve as reference for factory or tunnel fire safety designers and planners.
